# Supplementary material for: Comparative genomics provides new insights into the diversity, physiology, and sexuality of the only industrially exploited tremellomycete: Phaffia rhodozyma
Source: BMC Genomics. 2016 Nov 9;17:901. doi: 10.1186/s12864-016-3244-7 (PMC5103461; doi:10.1186/s12864-016-3244-7)
Supplement: Additional file 6: — List of orphan genes with links to PFAM (related to Additional file 1: Table S1). (ZIP 1428 kb) [file 12864_2016_3244_MOESM6_ESM.zip › BLAST_HTML_FTR/G02129_P.html]

BLAST Search Results


```
BLASTP 2.2.27+


Reference:
Stephen F. Altschul, Thomas L. Madden, Alejandro A. Schäffer,
Jinghui Zhang, Zheng Zhang, Webb Miller, and David J. Lipman (1997),
"Gapped BLAST and PSI-BLAST: a new generation of protein database
search programs", Nucleic Acids Res. 25:3389-3402.


Reference for
composition-based statistics:
Alejandro A. Schäffer, L. Aravind, Thomas L. Madden, Sergei
Shavirin, John L. Spouge, Yuri I. Wolf, Eugene V. Koonin, and
Stephen F. Altschul (2001), "Improving the accuracy of PSI-BLAST
protein database searches with composition-based statistics and
other refinements", Nucleic Acids Res. 29:2994-3005.


Database: nr
           71,551,133 sequences; 26,053,659,533 total letters


Query= G02129_P

Length=585
                                                                      Score     E
Sequences producing significant alignments:                          (Bits)  Value

emb|CED82146.1|  hypothetical protein [Xanthophyllomyces dendrorh...  1192    0.0  
gb|AKU99416.1|  Type IV fimbrial biogenesis protein PilY1 [Labili...  44.3    0.48 
ref|WP_050980330.1|  flagellar biosynthesis protein [Halomonas st...  41.2    4.0  
gb|KLJ08055.1|  hypothetical protein EMPG_16495 [Emmonsia parva U...  41.2    4.3  
gb|KOC17986.1|  cell polarity protein [Aspergillus flavus AF70]       40.8    5.1  
ref|XP_001209614.1|  hypothetical protein ATEG_06928 [Aspergillus...  40.4    6.2  
dbj|BAE60020.1|  unnamed protein product [Aspergillus oryzae RIB40]   40.4    6.5  
ref|XP_002379373.1|  cell polarity protein (Tea1), putative [Aspe...  40.4    6.6  
ref|XP_001822022.2|  cell polarity protein (Tea1) [Aspergillus or...  40.4    6.6  
gb|EIT78140.1|  kelch repeat-containing protein [Aspergillus oryz...  40.4    6.7  
gb|KJK60316.1|  hypothetical protein P875_00053469 [Aspergillus p...  40.4    7.2  
gb|KIL86712.1|  hypothetical protein FAVG1_09968 [Fusarium avenac...  40.0    8.4  
ref|XP_001272956.1|  cell polarity protein (Tea1), putative [Aspe...  40.0    8.9  


 >emb|CED82146.1| hypothetical protein [Xanthophyllomyces dendrorhous]
Length=586

 Score = 1192 bits (3083),  Expect = 0.0, Method: Compositional matrix adjust.
 Identities = 580/582 (99%), Positives = 581/582 (99%), Gaps = 0/582 (0%)

Query  3    VLATHARPNISVRSHSRSITNSLPFPPSRNVPAPRQMPLALPWETAQQLQSTGRSSRSIP  62
            +LATHARPNISVRSHSRSITNSLPFPPSRNVPAPRQMPLALPWETAQQLQSTGRSSRSIP
Sbjct  5    LLATHARPNISVRSHSRSITNSLPFPPSRNVPAPRQMPLALPWETAQQLQSTGRSSRSIP  64

Query  63   RYAEFKLNNTNLPAGGFQAYFDRMVKNKFELDDEAVMNVLRTFIGRYENETVGTKDDSLI  122
            RYAEFKLNNTNLPAGGFQAYFDRMVKNKFELDDEAVMNVLRTFIGRYENETVGTKDDSLI
Sbjct  65   RYAEFKLNNTNLPAGGFQAYFDRMVKNKFELDDEAVMNVLRTFIGRYENETVGTKDDSLI  124

Query  123  AIAKQIQTDGDDKVFPNMIHRSTSTKAIRSNTEGTLKDAMRVYRLILKQLKTIPAFDPVY  182
            AIAKQIQTDGDDKVFPNMIHRSTSTKAIRSNTEGTLKDAMRVYRLILKQLKTIPAFDPVY
Sbjct  125  AIAKQIQTDGDDKVFPNMIHRSTSTKAIRSNTEGTLKDAMRVYRLILKQLKTIPAFDPVY  184

Query  183  FSNEYNIDYNKKSWNRHFDAELAAMKQASSAQIRPIQERANEIADRLKVLIADGVSSAVE  242
            FSNEYNIDYNKKSWNRHFDAELAAMKQASSAQIRPIQERANEIADRLKVLIADGVSSAVE
Sbjct  185  FSNEYNIDYNKKSWNRHFDAELAAMKQASSAQIRPIQERANEIADRLKVLIADGVSSAVE  244

Query  243  ETQMLEAELRSSMKQLKELEMSLEESQQDVSEARKNKMLTLENDHRSFLSWMNGTIKKRR  302
            ETQMLEAELRSSMKQLKELEMSLEESQQDVSEARKNKMLTLENDHRSFLSWMNGTIKKRR
Sbjct  245  ETQMLEAELRSSMKQLKELEMSLEESQQDVSEARKNKMLTLENDHRSFLSWMNGTIKKRR  304

Query  303  ATLKKESDMFDSTRPTGLSNATTTNEIAEPSNLLHAVEKTVEFHKDQMFYQQLEMLYLAD  362
            ATLKKESDMFDSTRPTGLSNATTTNEIAEPSNLLHAVEKTVEFHKDQMFYQQLEMLYLAD
Sbjct  305  ATLKKESDMFDSTRPTGLSNATTTNEIAEPSNLLHAVEKTVEFHKDQMFYQQLEMLYLAD  364

Query  363  RFRQIALKINRPVLFKESFDLVVRTVTARRAPFNVSGPNTTLFQKVIKFAVARQLYRAMF  422
            RFRQIALKINRPVLFKESFDLVVRTVTARRAPFNVSGPNTTLFQKVIKFAVARQLYRAMF
Sbjct  365  RFRQIALKINRPVLFKESFDLVVRTVTARRAPFNVSGPNTTLFQKVIKFAVARQLYRAMF  424

Query  423  ALRSRSGSEADFKPLGPAIVYLEALLDLVKTQPDYILAKKTVLDVVSSVCENLDGATRRA  482
            ALRSRSGSEADFKPLGPAIVYLEALLDLVKTQPDYILAKKTVLDVVSSVCENLDGATRRA
Sbjct  425  ALRSRSGSEADFKPLGPAIVYLEALLDLVKTQPDYILAKKTVLDVVSSVCENLDGATRRA  484

Query  483  LLEPTTGLSGQSRSVSLETRVEPIAQFLRTAIEILKRSSSSAVASAETGLSKAQVERVSE  542
            LLEPTTGLSGQSRSVSLETRVEPIAQFLRTAIEILKRSSSSAVASAETGLSKAQVERVSE
Sbjct  485  LLEPTTGLSGQSRSVSLETRVEPIAQFLRTAIEILKRSSSSAVASAETGLSKAQVERVSE  544

Query  543  EVGHVRKEFVLEGAGSLVEVKAIGGSIKRDLEVLERAIKKLA  584
            EVGHVRKEFVLEGA SLVEVKAIGGSIKRDLEVLERAIKKLA
Sbjct  545  EVGHVRKEFVLEGADSLVEVKAIGGSIKRDLEVLERAIKKLA  586


>gb|AKU99416.1| Type IV fimbrial biogenesis protein PilY1 [Labilithrix luteola]
Length=1385

 Score = 44.3 bits (103),  Expect = 0.48, Method: Compositional matrix adjust.
 Identities = 29/95 (31%), Positives = 42/95 (44%), Gaps = 11/95 (12%)

Query  3    VLATHARPNISVRSHSRSITNSLPFPPSRNVPAPRQMPLALPWETAQQLQSTGRSSRSIP  62
            +L T   PN+++R+     T   P+P S     P     ++ W+ A+ L  TG    S+P
Sbjct  376  ILLTDGAPNLNMRTSCEGPTGQCPYPGS----TPTGSGQSIAWDIAKTLY-TGTGHHSVP  430

Query  63   RYA-EFKLNNTNLPAG-----GFQAYFDRMVKNKF  91
             +   F +N TN P G     GFQ   D    N F
Sbjct  431  VFVIGFSVNGTNSPTGDGFPAGFQTAPDNSCSNWF  465


>ref|WP_050980330.1| flagellar biosynthesis protein [Halomonas stevensii]
Length=710

 Score = 41.2 bits (95),  Expect = 4.0, Method: Compositional matrix adjust.
 Identities = 21/70 (30%), Positives = 36/70 (51%), Gaps = 6/70 (9%)

Query  267  ESQQDVSEARKNKMLTLENDHRSFLSWMNGTIKKRRATLKKESDMFDST------RPTGL  320
            E  Q+V EA  +++  L     + ++W+ G +  R + L++E   FD T       PTG+
Sbjct  111  ELSQEVVEALPDELAALPASSETLMTWLRGQLMARLSVLRQEDAFFDQTGIVALVGPTGV  170

Query  321  SNATTTNEIA  330
               TTT ++A
Sbjct  171  GKTTTTAKLA  180


>gb|KLJ08055.1| hypothetical protein EMPG_16495 [Emmonsia parva UAMH 139]
Length=1495

 Score = 41.2 bits (95),  Expect = 4.3, Method: Compositional matrix adjust.
 Identities = 21/53 (40%), Positives = 34/53 (64%), Gaps = 0/53 (0%)

Query  247   LEAELRSSMKQLKELEMSLEESQQDVSEARKNKMLTLENDHRSFLSWMNGTIK  299
             LE +L+SSMK  +E ++S E  +Q+   A   K+  LEND++S + ++ GT K
Sbjct  1156  LEQQLQSSMKAHEETKLSFESREQEADRAYNEKLEQLENDYQSAVHYVKGTEK  1208


>gb|KOC17986.1| cell polarity protein [Aspergillus flavus AF70]
Length=1885

 Score = 40.8 bits (94),  Expect = 5.1, Method: Compositional matrix adjust.
 Identities = 21/53 (40%), Positives = 34/53 (64%), Gaps = 0/53 (0%)

Query  247   LEAELRSSMKQLKELEMSLEESQQDVSEARKNKMLTLENDHRSFLSWMNGTIK  299
             LE +L +S+K  +EL+ S E  +Q+   A K K+  LEND++S + ++ GT K
Sbjct  1166  LEQQLSTSLKAHEELKASFETREQEADRAYKEKLEQLENDYQSAVHYVKGTEK  1218


>ref|XP_001209614.1| hypothetical protein ATEG_06928 [Aspergillus terreus NIH2624]
 gb|EAU32312.1| hypothetical protein ATEG_06928 [Aspergillus terreus NIH2624]
Length=1484

 Score = 40.4 bits (93),  Expect = 6.2, Method: Compositional matrix adjust.
 Identities = 20/53 (38%), Positives = 35/53 (66%), Gaps = 0/53 (0%)

Query  247   LEAELRSSMKQLKELEMSLEESQQDVSEARKNKMLTLENDHRSFLSWMNGTIK  299
             LE +L +S+K  ++L+MS E  +Q+   A + K+  LEND++S + ++ GT K
Sbjct  1156  LEQQLSASLKAHEDLKMSFETREQEADRAYREKLEQLENDYQSAVHYVKGTEK  1208


>dbj|BAE60020.1| unnamed protein product [Aspergillus oryzae RIB40]
Length=1523

 Score = 40.4 bits (93),  Expect = 6.5, Method: Compositional matrix adjust.
 Identities = 21/53 (40%), Positives = 34/53 (64%), Gaps = 0/53 (0%)

Query  247   LEAELRSSMKQLKELEMSLEESQQDVSEARKNKMLTLENDHRSFLSWMNGTIK  299
             LE +L +S+K  +EL+ S E  +Q+   A K K+  LEND++S + ++ GT K
Sbjct  1198  LEQQLSTSLKAHEELKASFETREQEADRAYKEKLEQLENDYQSAVHYVKGTEK  1250


>ref|XP_002379373.1| cell polarity protein (Tea1), putative [Aspergillus flavus NRRL3357]
 gb|EED50597.1| cell polarity protein (Tea1), putative [Aspergillus flavus NRRL3357]
Length=1491

 Score = 40.4 bits (93),  Expect = 6.6, Method: Compositional matrix adjust.
 Identities = 21/53 (40%), Positives = 34/53 (64%), Gaps = 0/53 (0%)

Query  247   LEAELRSSMKQLKELEMSLEESQQDVSEARKNKMLTLENDHRSFLSWMNGTIK  299
             LE +L +S+K  +EL+ S E  +Q+   A K K+  LEND++S + ++ GT K
Sbjct  1166  LEQQLSTSLKAHEELKASFETREQEADRAYKEKLEQLENDYQSAVHYVKGTEK  1218


>ref|XP_001822022.2| cell polarity protein (Tea1) [Aspergillus oryzae RIB40]
Length=1491

 Score = 40.4 bits (93),  Expect = 6.6, Method: Compositional matrix adjust.
 Identities = 21/53 (40%), Positives = 34/53 (64%), Gaps = 0/53 (0%)

Query  247   LEAELRSSMKQLKELEMSLEESQQDVSEARKNKMLTLENDHRSFLSWMNGTIK  299
             LE +L +S+K  +EL+ S E  +Q+   A K K+  LEND++S + ++ GT K
Sbjct  1166  LEQQLSTSLKAHEELKASFETREQEADRAYKEKLEQLENDYQSAVHYVKGTEK  1218


>gb|EIT78140.1| kelch repeat-containing protein [Aspergillus oryzae 3.042]
 gb|KDE79586.1| kelch repeat-containing protein [Aspergillus oryzae 100-8]
Length=1491

 Score = 40.4 bits (93),  Expect = 6.7, Method: Compositional matrix adjust.
 Identities = 21/53 (40%), Positives = 34/53 (64%), Gaps = 0/53 (0%)

Query  247   LEAELRSSMKQLKELEMSLEESQQDVSEARKNKMLTLENDHRSFLSWMNGTIK  299
             LE +L +S+K  +EL+ S E  +Q+   A K K+  LEND++S + ++ GT K
Sbjct  1166  LEQQLSTSLKAHEELKASFETREQEADRAYKEKLEQLENDYQSAVHYVKGTEK  1218


>gb|KJK60316.1| hypothetical protein P875_00053469 [Aspergillus parasiticus SU-1]
Length=1491

 Score = 40.4 bits (93),  Expect = 7.2, Method: Compositional matrix adjust.
 Identities = 21/53 (40%), Positives = 34/53 (64%), Gaps = 0/53 (0%)

Query  247   LEAELRSSMKQLKELEMSLEESQQDVSEARKNKMLTLENDHRSFLSWMNGTIK  299
             LE +L +S+K  +EL+ S E  +Q+   A K K+  LEND++S + ++ GT K
Sbjct  1166  LEQQLSTSLKAHEELKASFETREQEADRAYKEKLEQLENDYQSAVHYVKGTEK  1218


>gb|KIL86712.1| hypothetical protein FAVG1_09968 [Fusarium avenaceum]
Length=910

 Score = 40.0 bits (92),  Expect = 8.4, Method: Compositional matrix adjust.
 Identities = 48/176 (27%), Positives = 80/176 (45%), Gaps = 20/176 (11%)

Query  248  EAELRSSMKQLKELEMSLEESQQDVSEARKNKMLTLENDHRSFLSWMNGTIKKRRATLKK  307
            E+EL   +++L+ELE  LE S++  SE  +     LE+  +    +   T+    A L  
Sbjct  580  ESELERVVQELRELEPQLESSRRARSEVSEKVDANLEDTCKDIYDYTRNTLNS--AILHS  637

Query  308  ESDMFDSTRPTGLSNATTTNEI---AEPSNLLHAVEKTVEFHKDQMFY-----QQLEMLY  359
             S  +D   P G+ NA    E    A  S +  +V    ++ + +  Y     +QL +L+
Sbjct  638  GSSHYDVPYP-GIWNAFQYAEDLKEAMFSRIADSVSNCEDYARSKTVYGVNTIKQLGILH  696

Query  360  LADRFRQIALKINRP-VLFKESFDLVVRTVTARRAPFNVSG--PNTTLFQKVIKFA  412
            + D F+ +     RP V+F+   D + R V     P  ++     TTL Q+  KFA
Sbjct  697  VGDEFQNLQF---RPDVMFRRKRDALARQVD---IPTEMTDFVDWTTLLQRQEKFA  746


>ref|XP_001272956.1| cell polarity protein (Tea1), putative [Aspergillus clavatus 
NRRL 1]
 gb|EAW11530.1| cell polarity protein (Tea1), putative [Aspergillus clavatus 
NRRL 1]
Length=1502

 Score = 40.0 bits (92),  Expect = 8.9, Method: Compositional matrix adjust.
 Identities = 22/60 (37%), Positives = 39/60 (65%), Gaps = 2/60 (3%)

Query  242   EETQM--LEAELRSSMKQLKELEMSLEESQQDVSEARKNKMLTLENDHRSFLSWMNGTIK  299
             E+T++  LE +L +S+K  +EL+ S E  +Q+   A + K+  LEND++S + ++ GT K
Sbjct  1161  EQTRLRDLEQQLSTSLKAHEELKASFENREQEADRAYREKLEQLENDYQSAVHYVKGTEK  1220


Lambda      K        H        a         alpha
   0.317    0.130    0.356    0.792     4.96 

Gapped
Lambda      K        H        a         alpha    sigma
   0.267   0.0410    0.140     1.90     42.6     43.6 

Effective search space used: 6343016347056


  Database: nr
    Posted date:  Sep 23, 2015 12:05 AM
  Number of letters in database: 26,053,659,533
  Number of sequences in database:  71,551,133


Matrix: BLOSUM62
Gap Penalties: Existence: 11, Extension: 1
Neighboring words threshold: 11
Window for multiple hits: 40
```
